# The Application of Metabolomics in Frailty: Trends, Challenges, and Future Directions

**DOI:** 10.3390/metabo16060380

**Published:** 2026-05-31

**Authors:** Kaiying Fang, Bei Niu, Zhen Zhang, Yameng Jiang, Ya Zhao, Zhanguo Wang

**Affiliations:** School of Preclinical Medicine, Chengdu University, Chengdu 610106, China; 212025100100001@cdu.edu.cn (K.F.); niubei@cdu.edu.cn (B.N.); zhangzhen@cdu.edu.cn (Z.Z.); 212025105400009@cdu.edu.cn (Y.J.); 212025105400024@cdu.edu.cn (Y.Z.)

**Keywords:** metabolomics, frailty, hotspots, research trend, metabolic pathways

## Abstract

**Highlights:**

**What are the main findings?**

- Over the past two decades, the United States and China have led research output in metabolomics and frailty, with U.S. institutions ranking first in academic impact.
- Research focus has shifted from macro-level indicators such as inflammation and protein–energy wasting to amino acid, energy, lipid, and tryptophan metabolic pathways, as well as gut microbiota-derived butyrate and trimethylamine-N-oxide.

**What are the implications of the main findings?**

- A methodological framework integrating physiology, nutrition, geriatrics, and computational biology is provided, reusable for trend analysis in this field.
- Butyrate, trimethylamine-N-oxide, and tryptophan metabolites are identified as novel metabolic targets for early frailty detection and intervention.

**Abstract:**

Frailty is a geriatric syndrome involving inflammation, oxidative stress, mitochondrial dysfunction, and metabolic disturbances. Metabolomics can systematically elucidate metabolic pathways and identify actionable biomarkers. This study systematically reviews the progress and evolutionary trends of metabolomics applications in frailty research from 2006 to 2025. Based on 1924 publications retrieved from the Web of Science Core Collection, systematic analyses were performed using CiteSpace, VOSviewer, SCImago Graphica, and the R package “bibliometrix”, focusing on pathway-level research hotspots and collaboration networks. The United States and China are the leading contributors. Research hotspots have shifted from macro-level biomarkers such as inflammation and protein–energy wasting to specific metabolic pathways including amino acid metabolism, energy metabolism, lipid metabolism, and tryptophan degradation. Key metabolites include sphingomyelin, butyrate, and trimethylamine-N-oxide. Emerging frontiers focus on the association between gut microbiota-derived metabolites and frailty phenotypes, as well as intervention strategies targeting these metabolites. This study provides the first systematic overview of global research progress in metabolomics and frailty, establishes a reproducible evaluation framework integrating physiology, nutrition, geriatrics, and computational biology, and identifies butyrate, trimethylamine-N-oxide, and tryptophan metabolites as potential metabolic targets for early identification and intervention.

## 1. Introduction

Frailty is defined as a clinical syndrome in geriatric populations [1], characterized by reduced physiological reserve and impaired function across multiple systems [2]. Therefore, the person is more vulnerable to stressors and has an increased risk of negative health outcomes [3]. This pathological condition imposes a considerable disease burden worldwide [4]. Epidemiological studies have revealed marked heterogeneity in the global prevalence of frailty, ranging from 4.0% to 59.1% among community-dwelling older adults, with a pooled estimate of 17.4% [5]. The distribution in the population differs significantly; at the same time, it is higher in clinical settings [6]. It stood at 39.1% among hospitalized older adults [7], and ranged from 40% to 50% in cancer patients [8], a pattern particularly pronounced in countries with low and middle incomes [9]. There are still some gender differences, and the proportion of females is 29.0% versus 20.0% for males [10]. Frailty is also a relatively serious risk factor for serious illness, and it can significantly increase the risk of falls, disability, the need for medical institution admission, cognitive decline and death [11]. Furthermore, frail and pre-frail populations have a higher healthcare expenditure per person and place a greater burden on the healthcare system [12]. Amplified by population aging and rising global prevalence, frailty has emerged as a priority area for leading international health organizations such as the World Health Organization (WHO) and the International Association of Gerontology and Geriatrics (IAGG), necessitating a concerted public health response [9]. Two mainstream assessment models are currently adopted internationally: the Fried phenotype criteria, which comprise unintentional weight loss, weak grip strength, self-reported exhaustion, slow walking speed, and low physical activity, and frailty is diagnosed when three or more of these components are present [9]; and the cumulative deficit model, known as the Rockwood Frailty Index, which quantifies frailty as a continuous variable by calculating the proportion of accumulated health deficits, such as diseases and functional impairments, where an index of ≥0.25 is indicative of frailty [13]. Current diagnostic trends are shifting from the traditional phenotype and cumulative deficit models toward clinically oriented rapid screening tools such as the Clinical Frailty Scale (CFS) and multidimensional Comprehensive Geriatric Assessment (CGA), with the objectives of enabling earlier identification and guiding patient-centered, individualized intervention strategies [14]. To promote the early identification and targeted intervention of frailty, current research is dedicated to elucidating the roles of genetic, epigenetic, and multisystem dysregulation in the progression of frailty, aiming to pave new pathways for precision-based prevention and management in the future. Given that frailty fundamentally represents the depletion of multisystem physiological reserves, in-depth analysis of the underlying functional metabolic disturbances is key to linking clinical phenotypes with molecular mechanisms.

The integrated application of metabolomics in frailty research enables the systematic elucidation of pathophysiological mechanisms centered on metabolic dysregulation, identifies specific biomarker profiles and distinct metabolic signatures, and thereby provides crucial scientific evidence for the advancement of geriatric and translational medicine. Studies have demonstrated that the metabolic disturbances in frail individuals exhibit multidimensional features, involving systematic dysregulation in areas such as amino acid, energy, and lipid metabolism [15,16,17]. These disturbances are closely associated with core pathological processes such as mitochondrial dysfunction, oxidative stress, and chronic inflammation [18,19,20,21]. Recent influential reviews have deepened this understanding from multiple perspectives. For instance, Costa et al. [22] specifically examined the value of metabolism-related biomarkers, such as inflammatory markers and neuroendocrine indices in frailty risk stratification; Barros D. et al. [23] systematically elucidates the central role of dysregulated energy metabolism and explores the interventional potential of exercise-mediated myokines. Young ML et al. [24] revealed how alterations in the metabolic functions of the gut microbiota, such as through impacting the production of short-chain fatty acids, contribute to the development of frailty, and summarized related microbiota-based intervention strategies. Mishra M. et al. [13] elucidated the association between the dysregulation of specific pathways, such as amino acid metabolism and tryptophan degradation and frailty. Conforto R. et al. [25] highlighted the significance of body composition phenotypes, such as osteosarcopenic obesity and their associated metabolomic signatures, for guiding tailored nutritional interventions. At the translational level, metabolomics has driven significant advances: machine learning-based biomarker panels have enhanced the accuracy of early frailty identification [26], metabolic pathway analysis has provided novel targets for precision therapy [27], and multi-omics integration has systematically elucidated mechanisms such as the gut–muscle axis [28]. Devitt C. et al. [29] demonstrated that the specific metabolic pathway dysregulation revealed by metabolomics provides a foundation for integrating multimodal biomarkers and developing novel screening and intervention tools. Collectively, these research advances have deepened the comprehension of the multisystem dysregulation mechanisms underlying frailty, facilitating a shift in frailty management toward a mechanism-driven precision medicine paradigm.

To our knowledge, no bibliometric study has systematically analyzed the application of metabolomics in frailty. Given the significant role of metabolomics in frailty research and the lack of recent bibliometric investigations in this field, a systematic analysis is warranted. This study employs a systematic and visualized approach to examine metabolomics within frailty research, utilizing CiteSpace, VOSviewer, and Bibliometrix software for analysis, with the following objectives: (1) To map the thematic evolution and knowledge architecture of metabolomics research in the field of frailty from 2006 to 2025, thereby establishing a framework and evidence base for predicting future research directions. (2) Identify key contributors, leading institutions and collaborative networks, evaluate teams’ differentiated contributions in areas such as interdisciplinary research and technological development, to optimize resource allocation and foster collaborative innovation. (3) Quantitatively analyze application trends and scientific output efficiency in targeted and untargeted metabolomics and dynamically track convergence patterns and technical bottlenecks in emerging frontiers such as cellular metabolomics and microbiota–host co-metabolism, thereby providing empirical evidence for optimizing technical solutions. (4) Summarize research and clinical translation pathways for metabolic markers associated with frailty, and promote the establishment of clinical consensus and standards.

## 2. Materials and Methods

### 2.1. Data Sources and Search Strategy

The data for this bibliometric analysis were systematically retrieved from the Web of Science Core Collection (WoSCC) [30,31], a premier database for high-quality scientific publications. The complete, executable search string used in the Web of Science Core Collection (search date: 6 January 2026) is provided in Table S1 in the Supplementary Materials. The search included all publications in the database from the beginning until 31 December 2025, to cover the research comprehensively. The first search returned 1924 matching results. All retrieved records, including complete bibliographic information and cited references, were exported in plain text format to facilitate comprehensive analysis using specialized bibliometric tools [32]. To balance recall and precision, the search strategy combined standard metabolomics terms with broad synonyms, including metabolize and metabolism [33,34]. Broad terms were necessary to avoid missing the early literature (circa 2006–2012) published before the standard term metabolomics became widely adopted [34]. After the initial WoSCC retrieval (2082 records), a three-stage manual screening was performed: (1) title/abstract review to exclude records unrelated to metabolomics techniques; (2) application of strict inclusion/exclusion criteria, removing studies not directly relevant to metabolomics and frailty; and (3) cross-validation of reference lists of the final 1924 publications, confirming no key articles were omitted. This procedure follows the consensus that no software fully automates bibliometric data preprocessing [35], ensuring that all retained publications are directly relevant to the field.

### 2.2. Data Extraction and Quality Assessment

**Figure 1:**
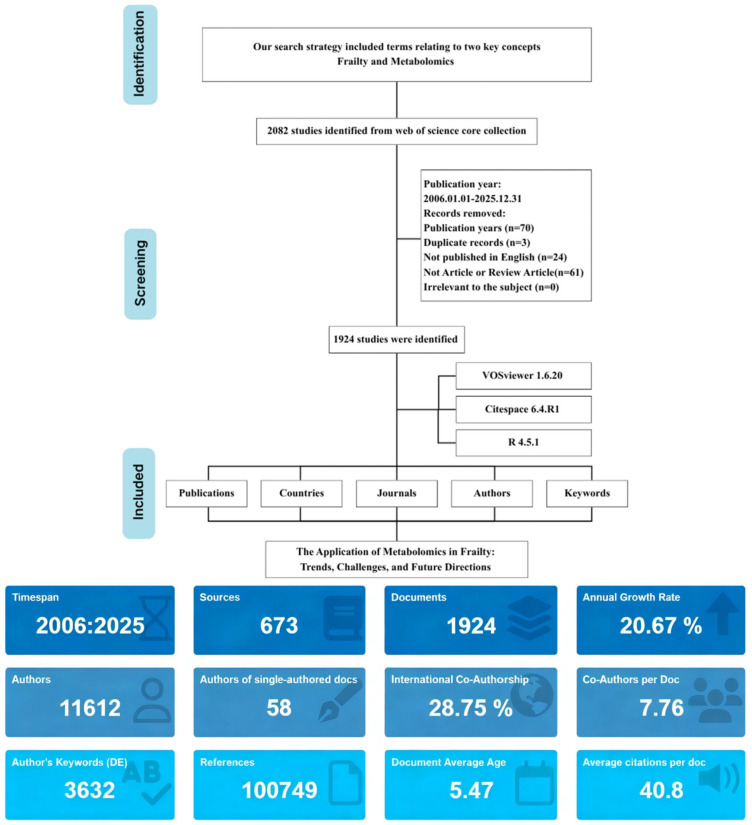
Flowchart of literature search strategy and selection process for metabolomics and frailty. Flow diagram of the bibliometric data screening process. After sequential exclusion by publication year (*n* = 70), duplicates (*n* = 3), non-English articles (*n* = 24), and document type (non-article/review, *n* = 61), a total of 1924 publications were obtained as the final analytical dataset. The figure shows “Publications: 1924” to reflect this number. It should be noted that upon initial import of the same dataset into the R package bibliometrix, the software temporarily generated a count of 1927. This minor discrepancy arose from the internal counting mechanism of the software when reading exported files and from routine statistical fluctuations during data preprocessing, which is consistent with the methodological consensus in bibliometric analysis. The number in the figure has now been standardized to 1924.

To ensure the reliability, accuracy, and reproducibility of the bibliometric analysis, this study adhered to a systematic literature retrieval, screening, and cleaning process. This process strictly followed predefined inclusion and exclusion criteria (refer to Figure 1). The initial screening of titles, abstracts, and keywords for all 2082 retrieved records was performed by a single reviewer based on the predefined inclusion/exclusion criteria. To assess inter-rater reliability, a second reviewer independently re-evaluated a random sample of 10% of the initial records (*n* = 208). The agreement on inclusion/exclusion decisions between the two reviewers was 98.6% (Cohen’s *κ* = 0.97), indicating excellent consistency. Disagreements were resolved through discussion [34]. A systematic cleaning of the raw data retrieved from the WoSCC was performed to address inconsistencies in keyword terminology, such as singular and plural forms, synonyms, and abbreviations. For instance, variants like metabolomics, metabolomic, metabonomics, and metabonomic were standardized to metabolomics. Similarly, terms related to frailty, such as frailty, frail older people, and physical frailty, were normalized. Data cleaning and terminology standardization were performed by a single reviewer following predefined rules based on MeSH terms and field consensus. The entire process was documented using the BIBLIO quality control table. A second reviewer independently performed a random sample check (10% of the final dataset) to verify the consistency of term standardization and inclusion/exclusion decisions, yielding 100% agreement. The above will guarantee the accuracy and comparability of the following keyword analyses. Literature inclusion criteria: (1) Document type: Original research papers and reviews; (2) Publication year: 1 January 2006–31 December 2025; (3) Language: English. Exclusion criteria: (1) Publications outside the time range [36], (2) Non-English publications, (3) Non-research document types, such as editorials, letters, conference abstracts, retracted publications and book chapters, (4) Studies that are not directly related to metabolomics and frailty, (5) Duplicate records and publications without accessible abstracts or full texts. A duplicate check was performed across all records, and no duplicates were identified within this final dataset. Following this screening process, a core dataset of 1924 high-quality publications (articles and reviews) was ultimately obtained, as depicted in Figure 1. It should be noted that this bibliometric analysis strictly followed standardized procedures in the field [37]. The sequential exclusion by publication year, duplicates, language, and document type was performed according to the methodological framework of Donthu et al. [33], and the cleaning and deduplication processes were guided by the data harmonization guidelines of Lim et al. [34]. Upon importing this dataset into the R package bibliometrix, the software automatically generated a document count of 1927. This minor discrepancy arises from the internal counting mechanism of the software when reading exported files, as also noted by van Eck and Waltman [38] regarding data parsing variations. As systematically reviewed by Błaszczyński and Karaś [35], such discrepancies in statistical granularity between phases are common and acceptable in bibliometric practice. The core analytical dataset for this study remains the 1924 publications, and all subsequent analyses were performed based on this dataset. The bibliometric quality control table (BIBLIO) (refer to Supplementary Materials Table S2) [39] was used to record all the steps of data collection, cleaning and validation, and ensure the openness and reproducibility of the analysis. The above organization of data ensured its accuracy and reliability for the subsequent bibliometric analysis.

### 2.3. Data Analysis and Visualization

Several analytical tools have been used to perform an all-encompassing bibliometric analysis, including CiteSpace (version 6.4.R1) [40], VOSviewer (version 1.6.20) [38], SCImago Graphica (version 1.0.53.0) [41] and the R package “bibliometrix” (version 5.4.0) [42] to facilitate a multi-dimensional examination and generate diverse visual mappings [43,44,45,46,47,48]. The selection of different bibliometric tools is based on the alignment of their methodological theories with the multidimensional analytical objectives of this study. CiteSpace employs burst detection algorithms and Kuhn’s theory of scientific revolutions [40,49] to identify critical turning points and research frontiers in frailty metabolomics, supporting the analysis of evolutionary trends over time. VOSviewer utilizes the VOS mapping technique and processes large-scale co-occurrence and citation coupling data through similarity matrices to reveal collaborative networks and thematic clustering structures, addressing the need for network dimension relationship mining [38]. SCImago Graphica relies on spatial statistics theory to geocode bibliometric indicators, presenting the distribution of publication outputs and collaborations across countries, thereby serving the global comparison of spatial dimensions [50]. The bibliometrix R package integrates classical models such as Lotka’s law and Bradford’s law [51,52] to provide comprehensive metrics including source impact, H-Index, and document coupling, while supporting reproducible statistical testing, covering performance assessment in the metric dimension [42]. Together, these four tools form a synergistic methodological chain across temporal, network, spatial, and metric dimensions, systematically analyzing the research structure and dynamics of frailty metabolomics. Subsequent to the initial retrieval of records from the WoSCC database, a rigorous data cleaning procedure was implemented. This process involved the standardization of bibliographic information and the systematic identification and removal of duplicate entries based on unique identifiers.

## 3. Results

### 3.1. Annual Publication Trends

**Figure 2:**
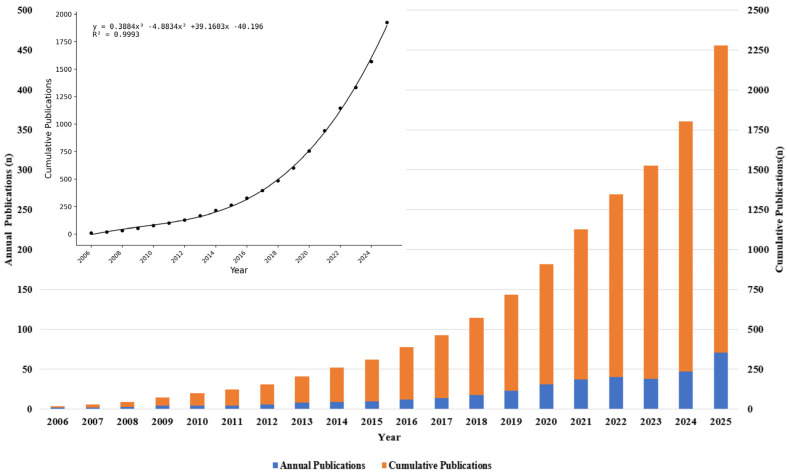
Annual and cumulative publication growth trends in metabolomics and frailty from 2006 to 2025. A cubic polynomial regression analysis yielded the equation *y* = 0.3884*x*^3^ − 4.8834*x*^2^ + 39.1603*x* − 40.196, with an *R*^2^ of 0.9993, indicating a strongly accelerated growth trend in cumulative publication output.

Annual publication volume and its evolutionary trend serve as a core basis for assessing the developmental pace and level of scholarly attention within a research field. Based on bibliometric data from 2006 to 2025, the publication activity in this research field demonstrates a significant phase-specific growth pattern (refer to Figure 2). Publication trend analysis reveals that the field has undergone three distinct developmental phases. The period from 2006 to 2012 marked a preliminary development phase, during which the annual publication output remained at a relatively low level of ≤28 publications and the cumulative publications totaled 127. From 2013 to 2018, the field entered a phase of steady growth, during which the annual publication output increased significantly from 40 publications in 2013 to 89 in 2018, with cumulative publications reaching 484 by 2018. Since 2019, the field has entered a phase of rapid expansion, with the annual publication output exceeding one hundred publications and maintaining continuous growth, reaching 235 publications in 2024 and further increasing to 355 in 2025. The cumulative annual publication output similarly demonstrated an exponential increase, rising from 10 publications in 2006 to 1924 publications in 2025. Quantitative assessment shows that the cumulative output in the research fields of metabolomics and frailty over the period 2006–2025 follows a cubic polynomial function (*y* = 0.3884*x*^3^ − 4.8834*x*^2^ + 39.1603*x* − 40.196) (refer to Figure 2), confirming that the field has established a sustained and accelerated developmental trajectory, characterized by a systematic expansion in research scale.

### 3.2. Countries/Regions and Institutions Co-Occurrence

**Figure 3:**
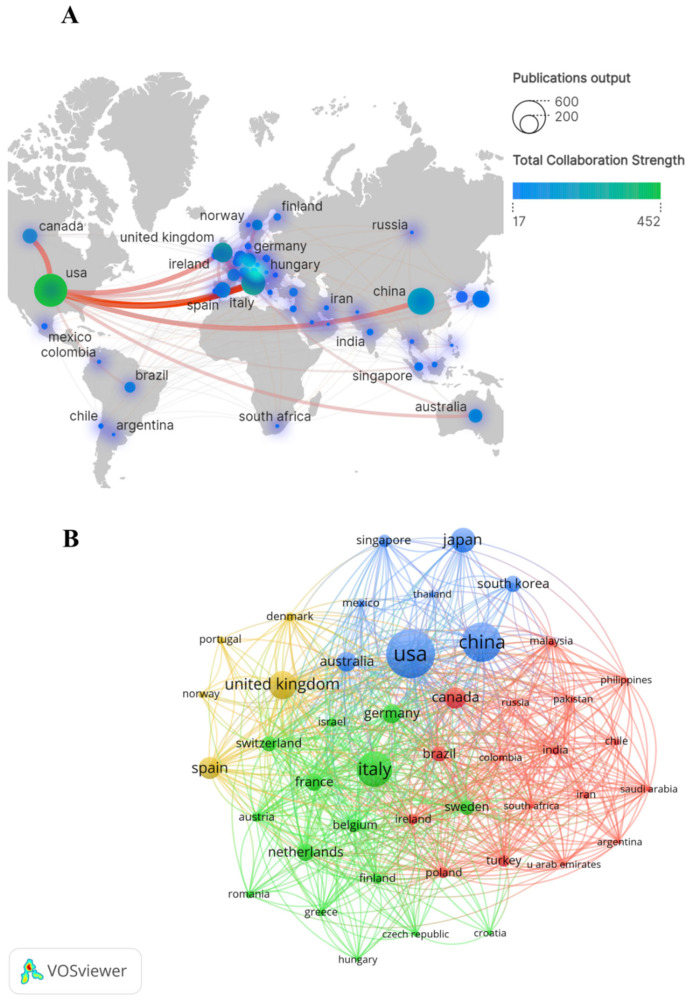
Global collaboration network in metabolomics and frailty research. (**A**) Geographical distribution map based on the total publications of different countries/regions. (**B**) The countries/regions’citation network visualization map was generated by using the VOSviewer. Different colors in the figure represent different clusters. The thickness of the lines reflects the citation strength.

**Figure 4:**
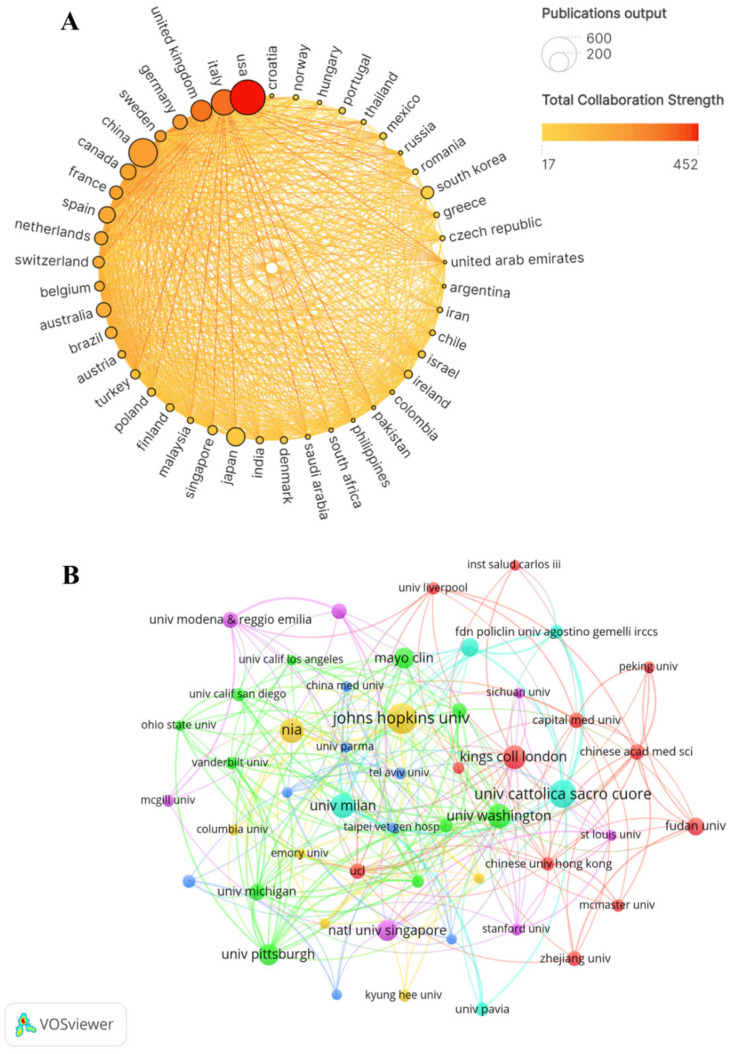
Institutional productivity in metabolomics and frailty research. (**A**) Visualization map of international collaborations by countries/regions. The size of the nodes represents the publication output, while the density of the connecting lines and the color of the nodes indicate the strength of collaboration between countries. (**B**) The institutions’ citation network visualization map was generated by using a VOSviewer. Different colors represent different clusters; within the same color cluster, institutions are more closely connected in terms of citation relationships.

Based on bibliometric data, the global research landscape in this field exhibits a clear hierarchical distribution and well defined collaborative patterns. Country- and regional-level data (refer to Table 1) show that the United States leads in total publication output with 548 papers and the highest H-Index of 89. However, when comparing research quality using average citations per paper, the United States ranks fifth among the top ten countries with a value of 59.77, lower than Australia at 87.05, Germany at 80.63, the Netherlands at 73.06, and England at 65.23. It should be noted that average citation per paper can partially normalize differences in publication volume, but it is also susceptible to being skewed by a small number of highly cited papers and may disadvantage recently published work that has not yet accumulated sufficient citations. China ranks second with 304 papers, with an average citation per paper of 30.84. Among the 304 publications from China, a total of 87 Chinese institutions contributed, with an average of approximately 3.5 publications per institution. The Netherlands, England, and Canada all have average citations exceeding 65, while Australia and Germany reach notably high values of 87.05 and 80.63, respectively. Overall, when comparing research quality across countries, it is advisable to consider multiple indicators together, including the H-Index, total citations, and publication volume. Institutional-level analysis (refer to Table 2) reveals a pronounced dominance of U.S. institutions, which occupy six of the top ten positions globally. The University of California System leads with 63 publications and 5769 total citations, with a notably high average of 91.57 citations per paper. Italy’s Catholic University of the Sacred Heart and IRCCS Policlinico Gemelli exhibit outstanding performances, averaging over 77 citations per paper and sharing an H-Index of 26, which exemplify the high-quality research profile of European institutions. Analysis of international collaboration networks further reveals a country/region-centered cooperative structure. The United States serves as the central hub of the global network (refer to Figure 3) and its internal institutions have formed closely connected collaborative clusters (refer to Figure 4B). China, as a rapidly emerging research entity, has been progressively expanding its international collaborative ties (refer to Figure 4A). European countries, meanwhile, have demonstrated a pattern of clustered regional multilateral cooperation (refer to Figure 3A), constituting another significant collaborative framework in this research domain.

### 3.3. Journals and Co-Cited Journals

**Figure 5:**
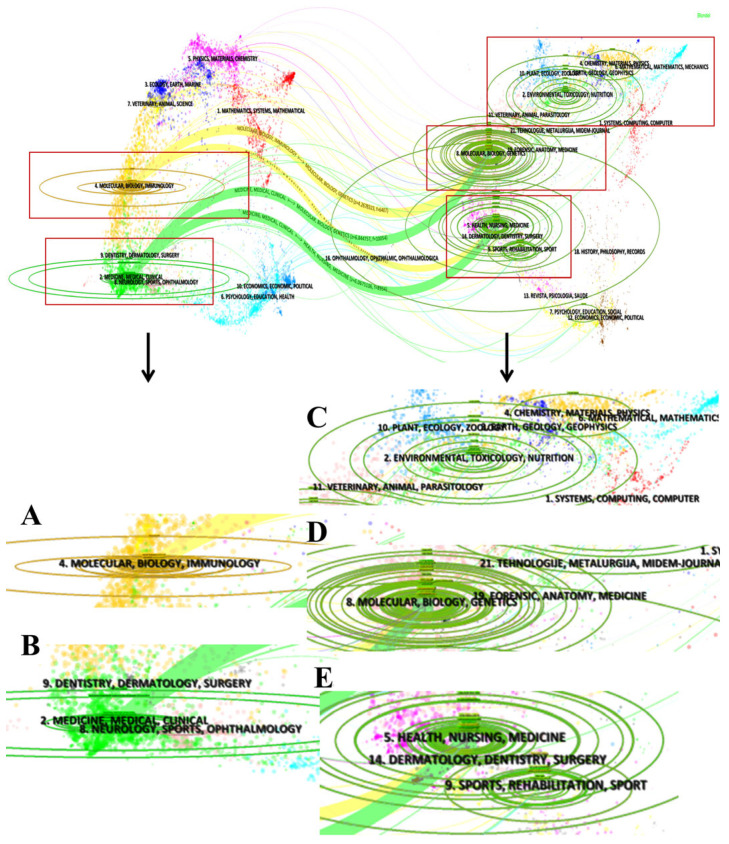
The dual-map overlay and corresponding disciplines. The citing journals are on the left, the cited journals are on the right, and the colored path represents the citation relationship. Different academic fields are represented by different color clusters; the lines between two color clusters represent the collaborative relationships between the research fields. (**A**) Disciplines represented in the citing journals: Molecular biology, immunology. (**B**) The journal clusters cover multiple medical and health sciences fields. (**C**) Disciplines in the citing or cited clusters. (**D**) Additional discipline clusters. (**E**) Disciplines with collaborative relationships.

**Figure 6:**
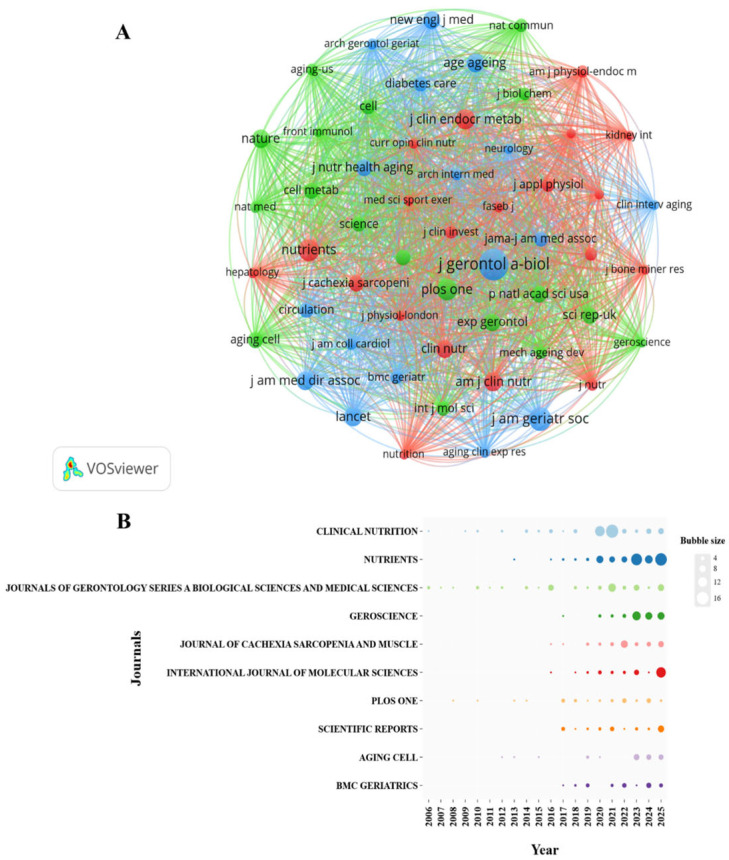
Journals and co-cited journals analysis. (**A**) Co-citation network of journals. Note: The colors (green, red, blue) represent distinct clusters formed by the co-citation analysis. (**B**) Bubble chart of annual publications for the top ten journals.

Analysis of publication and citation patterns in journals within the frailty and metabolomics domain reveals that knowledge production and dissemination in this field exhibit a distinct core–periphery structure and a notable characteristic of disciplinary concentration. Analysis of high-yield journals (refer to Table 3) indicates that research output is concentrated within a limited number of high-impact journals in the field of clinical nutrition. *Clinical Nutrition* ranks first with 78 publications, followed closely by *Nutrients* with 73 publications, highlighting the centrality of clinical nutrition and gerontology within this domain. In terms of impact, the *Journal of Cachexia*, *Sarcopenia and Muscle*, with a publication count of only 36 articles, holds the highest impact factor of 9.1 and Cite Score of 15.6, underscoring its authoritative standing in this specialized research subfield. Notably, open access (OA) journals have secured an absolute advantage in terms of total citations, with *Aging Cell* accumulating 834,622 citations and *PLoS ONE* 816,429 citations, far exceeding the citation volumes of traditional subscription-based journals. These citation counts for *Aging Cell* and *PLoS ONE* represent the global total citations of the journals across all subject areas in the Web of Science Core Collection, derived from the Journal Citation Reports (JCRs), and are not limited to the field of metabolomics and frailty. The disciplinary distribution of cited journals further reveals that the knowledge base of this field is highly concentrated at the intersection of physiology and nutrition, which closely aligns with the thematic focus of the high-yield journals identified (refer to Figure 5). Analysis of the journal collaboration network map (refer to Figure 6A) indicates that journals such as *Clinical Nutrition* and *Nutrients* form a nutrition–gerontology collaborative core, while open access journals occupy key positions in knowledge diffusion, consistent with their high citation metrics. In the bubble chart (refer to Figure 6B), the size of each bubble corresponds to the annual publication volume of a journal, categorized into four incremental size grades. After 2015, the majority of journal bubbles exhibit a pronounced enlargement, reflecting an overall increase in publication output across these journals while also revealing their heightened research activity within this field.

### 3.4. Authors and Co-Cited Authors

**Figure 7:**
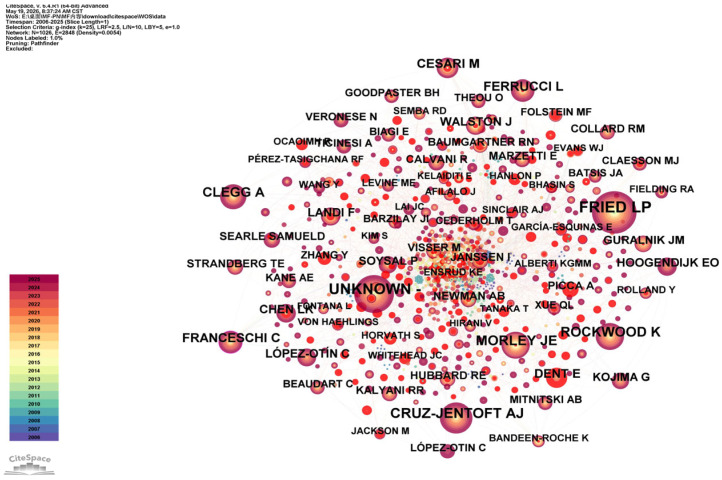
Cited authors co-occurrence network. Each node, distinguished by varying hues and sizes, represents an individual researcher. The size of a node is indicative of the number of publications, whereas the color signifies the year of publication.

The academic landscape of frailty and metabolomics research is characterized by a core group of authors who are both highly productive and closely interconnected. Analysis of highly productive authors (refer to Table 4) indicates that Marzetti, Emanuele is the scholar with the highest publication output, having authored 24 papers, which have accumulated 1722 total citations and an H-Index of 19. Notably, scholars such as Luigi Ferrucci, with 21 publications and an average of 99.33 citations per paper, and Landi, Francesco, with 18 publications and an average of 104.28 citations per paper, demonstrate exceptional research impact relative to their publication volume. A stable network of collaborative relationships exists among the key contributors, including Calvani, Riccardo and Picca, Anna. Citation network analysis (refer to Figure 7) further delineates this structure, where Cesari, Matteo serves as a core node connecting other key investigators including Luigi Ferrucci, Jeremy Walston, Riccardo Calvani, and Emanuele Marzetti.

### 3.5. Co-Cited Reference Analysis

**Figure 8:**
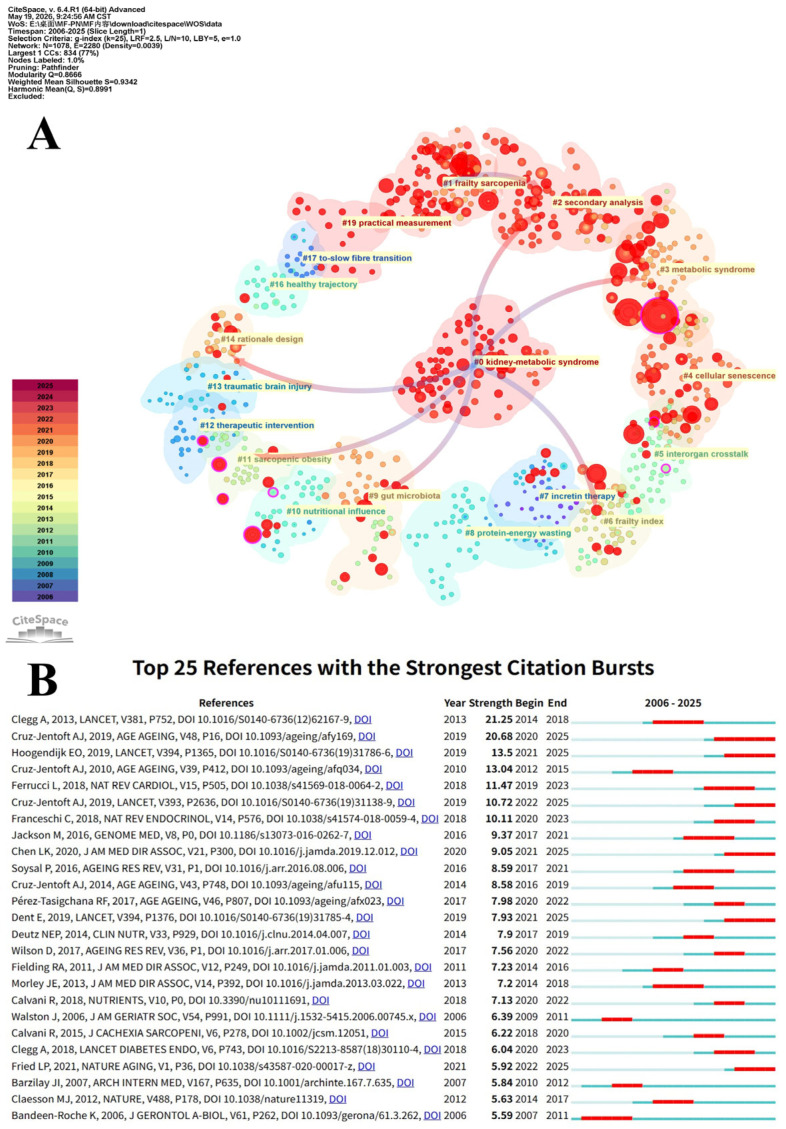
Co-cited reference analysis. (**A**) Timeline visualization of keyword clusters from 2006 to 2025, depicting the emergence and interaction of major themes over time. The purple circles indicate keyword nodes with high centrality, suggesting that these nodes serve as important bridges or hubs connecting multiple research clusters in the thematic evolution. The symbol “#” in the figure is an automatically generated prefix for cluster numbering by the software. (**B**) Top 25 references with the strongest citation bursts. The timeline reveals the field’s evolution from foundational phenotyping and consensus building to recent mechanistic and systems-level frameworks.

Literature co-citation cluster analysis (refer to Figure 8A) reveals that current research has consolidated into 18 core themes. Examination of the high-frequency and high-impact themes among them allows for the delineation of their interrelationships. Themes “frailty sarcopenia” and “skeletal muscle atrophy” focus on the pathophysiological intersection of frailty and sarcopenia. Themes “metabolic syndrome” and “kidney-metabolic syndrome” are related to metabolic dysregulation and multimorbidity. Themes “cellular senescence” and “interorgan crosstalk” explore mechanisms at the cellular and systemic levels. Themes “frailty index” and “practical measurement” are associated with the refinement of assessment tools and phenotypes. Theme “gut microbiota” reflects host–microbiota interactions. Themes “nutritional influence”, “therapeutic intervention” and “dietary intake” concentrate on nutritional and interventional strategies. Themes “urinary incontinence”, “healthy trajectory”, and “traumatic brain injury” represent research on specific health outcomes and life-course trajectories. This thematic structure is further corroborated dynamically by the burst analysis of co-cited references (refer to Figure 8B), which identified 25 publications exhibiting the strongest citation bursts. The development of the field originated in 2006–2007 with foundational studies on frailty and inflammation by Bandeen-Roche [53], burst strength 5.59; Walston [54], burst strength 6.39; and Barzilay [55], burst strength 5.84. This was followed by the period from 2010 to 2015, during which consensus guidelines on sarcopenia by Cruz-Jentoft [56], burst strength 13.04, and the systematic review by Clegg in *The Lancet* [57], burst strength 21.25, as the highest among all publications, established the core conceptual framework. In recent years, research frontiers have shifted toward systems biology. The 2016 paper by Jackson on the gut microbiota [58], burst strength 9.37, and the influential works on “inflammaging” by Ferrucci 2018 [59], burst strength 11.47, and Franceschi 2018 [60], burst strength 10.11, sustained their impact through 2023. Meanwhile, the updated consensus by Cruz-Jentoft 2019 [61], burst strength 20.68, and the 2020 guidelines by Chen [62] are driving the field toward precision and individualization.

**Figure 9:**
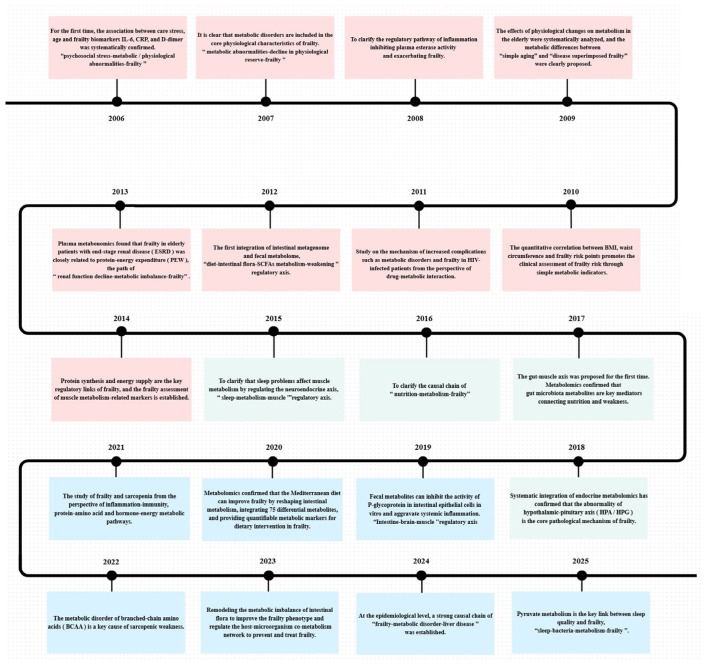
Historical timeline of key discoveries related to metabolomics and frailty. This timeline summarizes key advances in applying metabolomics to frailty research from 2006 to 2025. Early (2006–2014) [63,64,65,66,67]: Biomarker validation: Established metabolic dysfunction as central to frailty, validating biomarkers of systemic inflammation and oxidative stress. Middle (2015–2018) [68]: Mechanistic expansion: Elucidated tissue-specific pathways in muscle and gut microbiota, introducing the “gut–muscle axis” and the role of microbial metabolites. Recent (2019–2025) [69,70,71,72]: Systems integration and translation: Employs multi-omics to define core mechanisms and explores targeted interventions via microbial metabolism.

This progression is fully delineated in the research thematic timeline (refer to Figure 9). From 2006 to 2014, the focus was on foundational inflammatory markers and pharmacokinetics. During 2015 to 2018, research shifted toward the gut microbiome and dietary interventions, exemplified by emerging concepts such as the gut–muscle axis. From 2019 to 2025, research frontiers have advanced to include systematic reviews of shared biomarkers for frailty and sarcopenia, investigations into the effects of probiotics on muscle function, and analyses of frailty-associated risk in metabolic dysfunction-associated steatotic liver disease. This evolution marks the field’s entry into a new phase, characterized by the deep integration of multi-omics biomarker-driven mechanistic exploration and clinical interventions targeting gut microbiota and metabolic regulation. However, despite these advances, among the biomarker panels derived from metabolomics for frailty identified in the literature, the majority remain at the discovery or exploratory validation stage. Only a few have progressed to observational validation, but none have completed multistage clinical validation, established age- and sex-stratified reference ranges, or received formal consensus guidelines.

### 3.6. Keywords and Co-Occurrence Analysis

**Figure 10:**
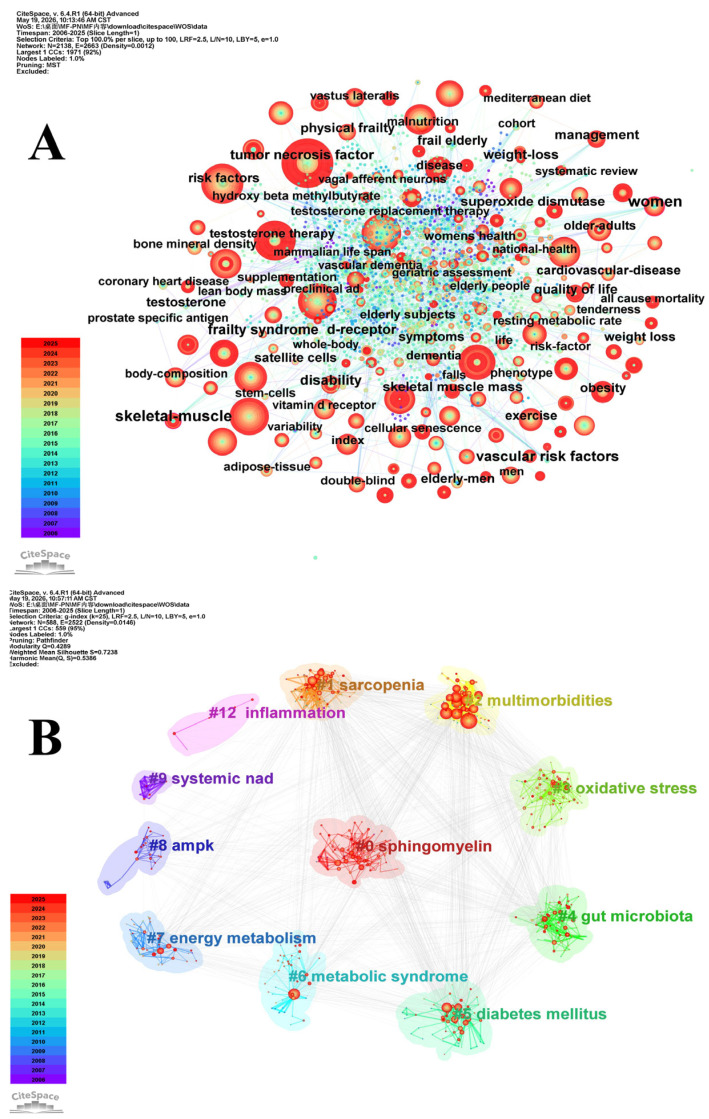
Thematic evolution and knowledge structure of frailty and metabolomics research from 2006 to 2025. (**A**) Keyword co-occurrence network and its structural metrics. High-frequency nodes are the key ideas of the field. The color gradient shows the average year of publication for the keywords, and warmer colors (red) are more recent. (**B**) Clustered keyword network. The analysis resulted in 11 distinct thematic clusters. The symbol “#” in the figure is an automatically generated prefix for cluster numbering by the software.

**Figure 11:**
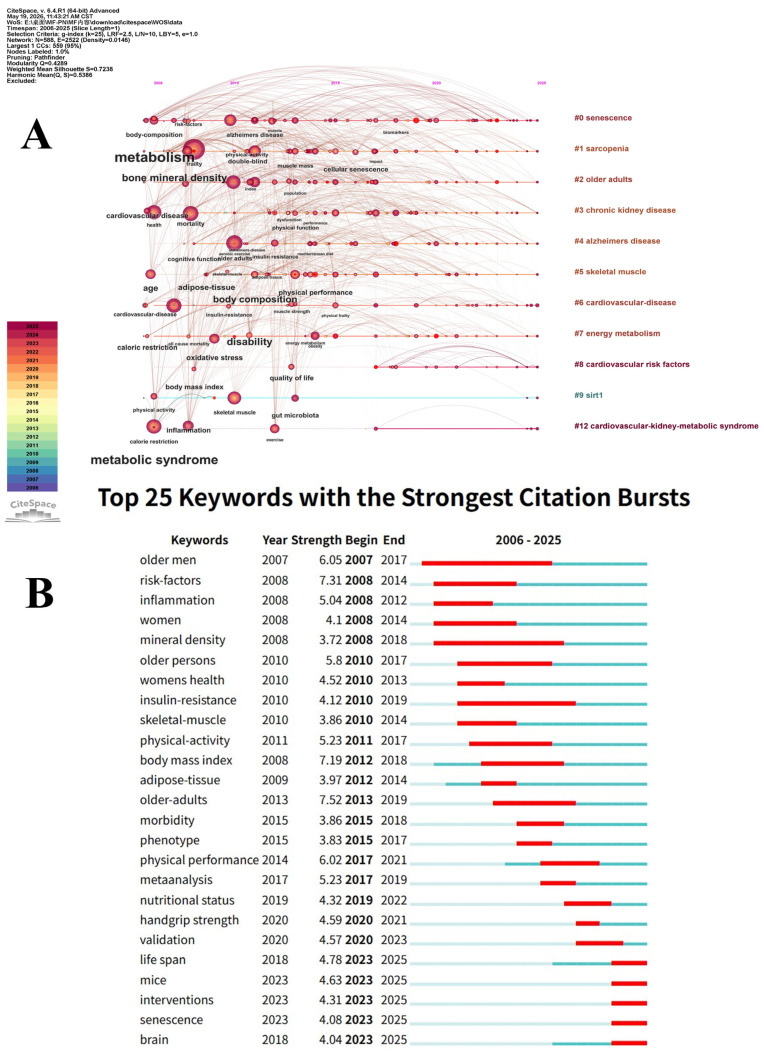
Thematic evolution and knowledge structure of frailty and metabolomics research from 2006 to 2025. (**A**) Timeline visualization of keyword clusters. Different colors represent different keyword clusters. The above figure shows the time-series evolution of the main keyword clusters from 2006 to 2025 and how research focus has changed over time. (**B**) The top 25 keywords with the largest citation bursts. Burst detection analysis finds words that have recently been used often and show a sudden rise in their mention frequency.

Through co-occurrence and burst analysis of domain keywords, this study systematically elucidates the core knowledge structure, thematic clusters, and temporal evolutionary trajectory of metabolomics and frailty research. The keyword co-occurrence network constructed using CiteSpace (refer to Figure 10A) exhibits a markedly modular structure, with high internal consistency observed within individual clusters. Cluster analysis revealed that the keywords formed 11 primary clusters (refer to Figure 10B). Core clusters, interpreted by the authors based on the automated clustering results from CiteSpace, such as gut microbiota, sphingomyelin, diabetes mellitus, metabolic syndrome, energy metabolism, AMPK, systemic NAD+, and inflammation, demonstrate a focused investigation into specific pathophysiological mechanisms. Among these, the metabolic syndrome node exhibits high centrality and connectivity, serving as a core hub in the research domain. By integrating the temporal evolution pattern of research priorities (refer to Figure 11A) and analyzing the 25 keywords with the highest citation burst strengths (refer to Figure 11B), a clear developmental trajectory is revealed. From 2006 to 2014, research primarily focused on macro-level risk factors, evidenced by the emergence of keywords such as “risk factors” with a burst strength of 7.31, “inflammation” at 5.04, and “body mass index” at 7.19. From 2015 to 2018, the main work has been to quantify phenotypes and collect findings; in this period, the top two were “physical performance” at 6.02 and “meta-analysis” at 5.23. Research shifted from directions to mechanisms and targeted therapies between 2019 and 2025. Keywords “mice” with a burst strength of 4.63, “interventions” at 4.31, “senescence” at 4.08, and “brain” at 4.04 have been established as the current frontiers. It can be seen from the above that the field of study has begun to develop along new paths, and animal models will be used to prove the mode of action to find cures for cell aging and nerve problems.

## 4. Discussion

### 4.1. Analysis of the Main Findings

This study presents the first comprehensive analysis of the world’s research on the application of metabolomics in frailty. Over the past two decades, the number of papers published and the frequency of citations have both increased significantly since 2019. This indicates that researchers around the world are also paying more attention to this problem and investing a lot of funds in research, but there is still a lack of precision prevention and treatment strategies. The annual growth rate of 20.67% indicates a rapidly expanding research interest in metabolomics applied to frailty. Meanwhile, only 58 publications are single-authored, suggesting a highly collaborative nature of this interdisciplinary field. Analysis shows that the United States is the leading country in the world for both paper output and average citation frequency per scholar, thus taking the lead in metabolomics research on frailty. It is in line with the whole country’s long-standing will to advance medical research and development and the strategic priority of disease prevention and control for an aging population.

Geographically, the United States maintains a leading position in research contributions, while China has rapidly increased its output. This reflects the substantial resource commitment and academic dynamism in both nations and indicates that China is at a critical transition from quantity accumulation to quality enhancement. Continued efforts are required to further elevate the impact and quality of its research outcomes. This pattern of “high output but low impact” may be influenced by multiple factors. First, the research evaluation system in China has long prioritized the number of SCI-indexed papers, which has driven rapid growth in publication volume but may also have encouraged following research hotspots rather than pursuing original innovation. Second, language barriers and the limited coverage of domestic journals in the Web of Science Core Collection may affect the international visibility and citation accumulation of some research outputs. Third, domestic collaboration still dominates, while international collaboration remains at an expanding stage; the relatively high homogeneity among domestic institutions may restrict cross-cultural and cross-disciplinary intellectual exchange, thereby affecting innovation and academic influence. These factors may partly explain China’s research performance in this field. Nevertheless, its overall contribution and upward trajectory are evident. With continued reform of the evaluation system and deeper international collaboration, the observed gaps are expected to gradually diminish. Notably, no institution from China appears among the top ten publishing organizations, suggesting a relatively dispersed distribution of research output across many Chinese institutions rather than concentration in a few. The regionalization pattern observed in international collaboration networks highlights a critical opportunity to strengthen cross-border partnerships. Collaboration networks can accelerate the discovery process by fostering diverse perspectives and shared research resources, particularly in fields increasingly reliant on complex technologies and interdisciplinary approaches. Future strengthening of quality-driven dialog and collaboration, with the United States and China as central hubs and European countries forming secondary clusters, will be essential to support emerging forces in achieving high-quality development, enhance the field’s overall innovative capacity, overcome current challenges, and advance the discipline toward more impactful outcomes.

An extensive analysis of author collaboration networks reveals a tightly knit collaborative structure with a high clustering coefficient, centered around a few highly productive authors who act as key hubs in the network, such as Emanuele Marzetti [73,74,75,76] and Matteo Cesari [77,78,79]. Among them, Emanuele Marzetti [70] has achieved pivotal progress in the area of geriatric frailty and sarcopenia, conditions with substantial clinical overlap, through large-scale systematic reviews and meta-analyses, thereby elucidating their shared biological foundations for subsequent research. By integrating multinational data and employing a multi-marker analytical strategy, Emanuele Marzetti has effectively addressed traditional limitations such as the limited explanatory power of single biomarkers and population heterogeneity. Thus, a series of key shared biomarkers has been systematically identified, such as hypoalbuminemia, low hemoglobin, and age-related changes in IL-6 and TNF-α; together, these indicate underlying mechanisms for multisystem dysregulation, including chronic inflammation, metabolic disturbances, and reduced hematopoietic function. Emanuele Marzetti has provided the measurable, objective biological basis for both conditions, as well as a strong evidentiary foundation for the future development of integrated diagnostic systems and targeted intervention strategies; thus, this research has extended the depth and prospects of frailty biomarker studies. Therefore, this author and his research team can reasonably be expected to continue producing highly influential research in the future. Furthermore, leading scholars in this field not only exhibit substantial individual output and notable influence but also, through stable collaborative relationships, drive the rigorous validation and theoretical refinement of key hypotheses such as “inflammaging associated frailty” and “energy metabolism dysregulation”. By concentrating on pivotal scientific questions, they promote efficient and high-quality knowledge production within the discipline. Matteo Cesari [80], grounded in a multisystem physiological integration perspective within geroscience, utilized a World Health Organization, convened cross-regional expert consensus meeting to integrate key biological mechanisms of frailty including chronic low-grade inflammation, mitochondrial dysfunction, and energy metabolism imbalance with observable clinical and functional phenotypes. This work led to the first definition of “intrinsic capacity” and its candidate biomarkers, established that declining intrinsic capacity constitutes a precursor state of frailty, and innovatively proposed dynamic proactive monitoring of intrinsic capacity for early identification and intervention in frailty. Matteo Cesari and his group have established a new consensus that is set to redefine the direction of future research on frailty; that is to say, it will be preventive rather than treatment-based and focus on maintaining functional ability. It will offer a foundation for developing assessment tools at both the individual and population levels, and is expected to help guide clinical practice, inform public health policies, and lead future health intervention research.

However, this collaboration network also exhibits a dual nature. In journal analysis, most of the research results have been concentrated in a small number of traditional core journals for clinical nutrition and gerontology, such as *Clinical Nutrition* [81,82]. Therefore, this field has achieved good clinical potential and a nutritional focus, and it is also one of the high-impact areas for this author group. The *Journal of Cachexia*, *Sarcopenia and Muscle* [71,83,84,85,86,87], with an extremely high impact factor, it has become the leading platform for deep studies of mechanisms in specific directions, such as frailty and sarcopenia, and leads front-line research. Although resources has been very focused, there is also the risk of homogeneity among research ideas. It will harm the continuous development of research innovation and high-quality development for metabolomics and frailty studies. Interestingly, open access (OA) journals such as *Aging Cell* [88,89,90,91,92,93,94,95], *PLoS ONE* [96,97,98,99,100,101], and *International Journal of Molecular Sciences* [102,103,104] exhibit a dominant advantage in total citation counts. It can be seen that the OA model has indeed been applied to spread knowledge quickly and widely, and at the same time, it also suggests a future direction of more diverse and dynamic indicators for evaluating academic influence. Beyond the accessibility effect noted above, the substantial citation advantage of open access journals may also be shaped by the type and depth of research they publish. The high total citation counts of OA journals such as *Aging Cell* and *PLoS ONE* likely result from the interplay of two mechanisms. First, the OA model lowers access barriers, broadens readership, and accelerates knowledge dissemination, thereby increasing the likelihood of citation. Second, there are systematic differences in the type and depth of research published across journals. *Aging Cell* specializes in mechanistic studies of aging, and such hypothesis-driven, methodologically rigorous work has a high citation potential regardless of its OA status. *PLoS ONE*, while covering a much wider disciplinary scope, also benefits from its large publication volume and the general OA effect. Therefore, the citation advantage of OA journals cannot be attributed solely to accessibility; research quality, topical relevance, and journal positioning are equally important. Future studies using matched comparisons or multivariate regression analyses are needed to disentangle the OA effect from content-driven citation impact. The disciplinary distribution of cited journals further indicates that the knowledge base of this field is firmly rooted in the interdisciplinary convergence of physiology, nutrition, and geriatrics. Such a solid interdisciplinary foundation serves as a crucial guarantee for its sustained innovation and development. While maintaining the existing high-quality collaborative ecosystem, future development of the field must consciously foster the integration of new talents across teams, regions, and especially from interdisciplinary backgrounds such as computational biology and clinical medicine, in order to stimulate a broader innovation landscape. To mitigate the risk of research homogenization in frailty metabolomics that may be driven by a small number of core journals, interdisciplinary teams may adopt two complementary strategies. First, computational biologists and geriatricians are encouraged to follow the CURE transparency principles [105] by pre-registering data processing workflows at the study design stage and using the CRediT taxonomy [106] to clarify contributor roles, thereby enhancing reproducibility and methodological cross-fertilization. Second, it is advisable to share key methodological results or negative findings ahead of formal publication through preprint servers such as bioRxiv/medRxiv or open access journals, taking advantage of the open access citation advantage to obtain interdisciplinary feedback before publication, thereby diluting the concentration of academic discourse in core journals [107]. These voluntary measures, grounded in the current scholarly ecosystem, offer a feasible path toward a more open and innovative research system.

Co-citation relationships reflect the foundational knowledge of a specific field. In 2013, Clegg A et al. [57] published the article with the highest burst strength and greatest impact, a study in *The Lancet* that provided a comprehensive overview of the definition, pathophysiological mechanisms, assessment models, clinical and public health significance, and intervention strategies for frailty in older adults. This work first explicitly advocated for incorporating frailty identification into routine clinical decision-making. The research with the second-highest burst strength is the revised European consensus on the definition and diagnosis of sarcopenia, published by Cruz-Jentoft AJ et al. [61] in *Age and Aging* in 2019. Serving as one of the core physiological foundations of the frailty phenotype, this consensus updated the definition of sarcopenia by establishing low muscle strength as a central diagnostic criterion, while also providing a clinical operational pathway with specific cut-off values. This advancement contributes to improving the consistency of clinical identification and promotes early intervention. The decline in burst strength of highly cited references reflects a shift in research focus. For example, the frailty review by Clegg et al. (2013) [57] in *The Lancet* showed a burst strength of 21.25 from 2014 to 2020, after which it ceased. This transition suggests a paradigm shift from the establishment of conceptual frailty frameworks toward mechanistic explorations such as metabolomics and inflammatory pathways. Once foundational knowledge becomes conventional wisdom, it no longer registers as a research front, which aligns with the recent emergence of burst keywords like “gut microbiota” and “metabolomics”.

A further integration of the mechanistic links between metabolomics and frailty is warranted. Recent reviews have systematically summarized differences in circulating metabolomic profiles between frail and non-frail older adults, including alterations in amino acids, lipids, glycoprotein acetylation, and gut microbiota-derived metabolites such as short-chain fatty acids [13]. Synthesizing the available evidence, three interconnected mechanistic pathways connect these metabolite signatures to the frailty phenotype.

The first pathway involves chronic low-grade inflammation. IL-6, CRP, and GlycA show consistent associations with the frailty index across multiple cohorts, and Mendelian randomization studies have provided initial clues for a causal link between GlycA and frailty, although such studies remain limited. Current evidence is largely cross-sectional, and the direction of causality still requires longitudinal follow-up and further genetic analyses to confirm [13]. The second pathway is mitochondrial dysfunction and energy metabolism imbalance. Frail older adults exhibit restricted glycolytic and mitochondrial energy production, leading to increased reliance on the ATP–phosphocreatine system, which directly contributes to muscle weakness, slow gait speed, and loss of lean mass. Unfortunately, this conclusion derives primarily from cross-sectional data and lacks support from long-term dynamic observations [16]. The third pathway is the gut microbiota–metabolite–immune axis. Alterations in gut microbial composition may participate in frailty progression through inflammation and energy homeostasis, but direct human evidence remains limited.

Notably, compared with adjacent research fields, the metabolomics–frailty landscape revealed in this study exhibits clear specificity. Bibliometric analyses focusing on sarcopenia (exercise and nutrition) treat frailty as a secondary cluster [108], whereas frailty remains central in our study. Frailty–sarcopenia research often highlights shared pathways such as AMPK and cellular senescence [109], while our work emphasizes a metabolite-resolved network. Aging-related bibliometric studies typically center on oxidative stress and neurodegenerative diseases [110], yet our study underscores clinical translation pathways. These comparisons further confirm the distinctiveness of metabolomics–frailty research, characterized by its metabolite-level resolution and translational orientation.

In summary, although existing research has outlined multiple potential mechanistic pathways, causal inference, standardization of workflows, and consideration of population heterogeneity remain core bottlenecks limiting progress in the field. Therefore, moving metabolomics from association discovery toward clinical translation requires prioritizing these challenges. Specifically, efforts should focus on conducting large-scale multicenter longitudinal cohort studies combined with causal inference methods such as Mendelian randomization [16]. At the same time, there is an urgent need to promote standardized metabolomic detection and data analysis procedures to enhance cross-study comparability. Furthermore, attention should be paid to evaluating sets of biomarkers and integrating laboratory, clinical, and imaging indices [20], as well as accelerating the translation of effective metabolic interventions from animal models to human clinical practice [13]. Through these coordinated efforts, metabolomics holds promise for providing a more solid scientific foundation for the precision prevention and individualized treatment of frailty.

### 4.2. Hotspots and Frontiers

Keywords reveal the core content and evolutionary trends of research, thereby offering crucial insights into current hotspots and recent advances in the field; the timeline visualization illustrates the formation process of thematic clusters. After integrating similar keywords, the resulting keyword grouping and cluster analysis indicate that terms such as “sphingomyelin,” “energy metabolism,” “gut microbiota,” and “inflammation” occur frequently, while “metabolic syndrome” acts as a central hub within this research field [111,112,113,114,115], tightly linking multiple clusters. The trend in research focus is discernible from both the evolution of representative findings in the timeline visualization and the emergence of keyword bursts, indicating that research on frailty and metabolomics can be divided into three developmental phases.

The initial phase, spanning 2006 to 2014, focused on employing metabolomics to validate and establish the associations between macro-level biomarkers and frailty phenotypes [116,117,118], specifically investigating which metabolite alterations accompany frailty. The appearance of the burst term “inflammation”, “risk factor” and “body mass index” is consistent with the pattern shown in the timeline visualization. Empirical evidence indicates that inflammation and mitochondrial dysfunction are associated with frailty, and both are related to alterations in amino acid and energy metabolism and increased oxidative stress, and lipid metabolism. Roland et al. reported that high levels of inflammatory markers in the blood, such as interleukin-6, C-reactive protein and D-dimer, are caused by persistent, mild inflammation and coagulation activation that speed up the process of frailty progression. The research team led by Chul [119] further revealed that protein–energy wasting (PEW) is a main Patho mechanism of frailty. Inflammation is also likely causing the breakdown of muscle proteins directly, and excessive reactive oxygen species in the face of oxidative stress have led to protein oxidation and other damage to cells. A negative protein–energy balance directly depletes muscle mass, impairs muscle quality and function, creates a vicious cycle, and thereby intensifies the frailty phenotype in patients. Meanwhile, findings from the English Longitudinal Study of Ageing (ELSA) [118] revealed that risk factors can be associated with other features of frailty. Factors such as body mass index and abdominal obesity are associated with changes in the system of inflammation and oxidative stress, and dysmetabolism of lipids, including elevated triglycerides and low HDL cholesterol, that occur in the presence of visceral fat accumulation, which together can worsen insulin resistance and metabolic syndrome to accelerate the development of organismic frailty. Looking back at the first phase, the key progress and remaining challenges can be summarized as follows. This phase successfully established preliminary associations between frailty and inflammation, oxidative stress, and various metabolites, providing important clues for subsequent mechanistic studies. It is worth acknowledging that these exploratory efforts revealed the potential of metabolomics in frailty research. At the same time, limited by the knowledge and technical conditions at the time, most studies adopted cross-sectional designs, and causal relationships still require longitudinal data to confirm. Differences in metabolomic detection platforms and analytical workflows also influenced the comparability of results to some extent. These experiences provide valuable references for optimizing study designs and promoting standardization in future research.

From 2015 to 2018, research entered a new phase focused on mechanistic pathways of frailty, with the emphasis shifting toward exploring the metabolic regulatory axes that connect different organs and systems. Scholars aimed to elucidate how metabolic alterations contribute to frailty through specific physiological pathways [120,121]. During this period, core concepts such as the “hypothalamic–pituitary–adrenal/gonadal axis” and the “nutrition–metabolism–frailty causal chain” emerged in succession. Research by the Piovezan team revealed that within the hypothalamic–pituitary–adrenal/gonadal axis, a state of chronic hypercortisolism and reduced testosterone levels promotes muscle catabolism. This leads to muscle atrophy, diminished muscle mass and function, and consequently accelerates the progression of frailty. Furthermore, Ticinesi et al. [122], through randomized controlled trials and animal studies, demonstrated that nutrients shape the intestinal environment and influence metabolite production. High-protein, high-fat diets increase intestinal permeability and systemic inflammation, whereas Mediterranean and high-fiber diets enhance the body’s anti-inflammatory capacity and improve metabolic and muscle health. These findings laid crucial groundwork for subsequent research focused on preventing or delaying frailty by targeting the gut microbiota. Turning to the second phase, several conceptual and methodological advances deserve attention. This phase expanded the research focus from single metabolites to multisystem regulatory networks, introducing important concepts such as the hypothalamic–pituitary–adrenal/gonadal axis and the nutrition–metabolism–frailty causal chain. Randomized controlled trials and animal studies provided initial evidence for the effects of dietary interventions on the gut microenvironment and metabolic pathways. These advances have significantly enriched our mechanistic understanding of frailty. It should be noted, however, that most intervention studies at that time used intermediate markers as endpoints, with relatively limited long-term follow-up of the frailty phenotype itself. The temporal relationship between gut microbiota and frailty also remains to be further clarified. These limitations laid the groundwork for the next phase, which focused on targeted metabolic interventions.

From 2019 to 2025, research in this field has further deepened, with the focus gradually expanding to targeted metabolic pathways and precision interventions. Scholars are dedicated to investigating whether and how specific metabolic pathways can be intervened upon to reverse frailty [123]. In addressing current challenges, research has proposed remodeling gut microbiota metabolism to ameliorate frailty phenotypes. The gut microbiota of frail older adults is characterized by a decrease in butyrate producing bacteria, which possess anti-inflammatory and gut barrier enhancing properties, and a reduction in Prevotella species associated with dietary fiber metabolism and glucose regulation. Concurrently, there is an increase in pathobionts linked to chronic inflammation, insulin resistance, and cardiovascular risk. This overall decline in microbial diversity and disruption of microbiota structure, alterations in core taxa closely associated with frailty, often directly or indirectly accelerate frailty by affecting inflammation, metabolism, and intestinal barrier function. Therefore, Young et al. [24] indicated that the gut microbiota is a modifiable intervention target. Probiotics, prebiotics, and synbiotics can increase beneficial bacteria, reduce inflammatory markers, and improve cognitive and immune function. Dietary interventions also constitute an important strategy for modulating the microbiota to prevent or ameliorate frailty. The Mediterranean diet can increase butyrate-producing bacteria and Prevotella abundance, thereby mitigating frailty. Polyphenol-rich foods may improve frailty by enhancing gut barrier integrity and modulating microbiota composition. However, it should be noted that the effects of high-protein diets are heterogeneous and may elevate harmful metabolites such as trimethylamine-N-oxide (TMAO) [124]. Therefore, future research directions are likely to focus on longitudinal studies establishing causality for personalized dietary regimens or probiotic formulations based on individual microbiota profiles, strain-level metagenomic analyses, clinical trials targeting specific beneficial bacteria or metabolites, and the development of tailored intervention strategies. Regarding this phase, research is clearly shifting towards causal inference and precision intervention. Gut microbiota-derived metabolites have been established as modifiable intervention targets, and causal inference methods such as Mendelian randomisation have begun to be introduced, methodologically addressing the limitations of previous association studies. Personalized nutrition strategies based on individual microbiome profiles have shown promising prospects. However, current intervention studies generally have short follow-up periods, and the heterogeneous effects of strategies such as high-protein diets suggest the need for more detailed subtype stratification. Furthermore, standardization of metabolomic detection workflows has not yet been widely implemented in the field, and cross-cohort data integration still has room for improvement. Future large-scale multicenter longitudinal studies combining multi-omics and causal inference tools are expected to further validate the clinical value of targeting specific metabolic pathways.

Despite the growing body of evidence linking metabolomics to frailty, its clinical translation remains a major challenge. A recent systematic review concluded that most candidate metabolite panels lack multistage validation, with the absence of standardized pre-analytical protocols being the primary bottleneck [125]. A multicenter study based on multiple cohorts from UK Biobank systematically analyzed 168 NMR metabolomic biomarkers in relation to the frailty index and identified 59 independently associated metabolites. Among these, the causal link between glycoprotein acetylation, an inflammatory marker, and frailty was particularly notable. However, the metabolite panel identified in this study has not yet been developed into a standardized clinical panel with age- and sex-stratified reference ranges [93]. An international Delphi consensus selected 14 aging biomarkers covering inflammatory, functional, and metabolic domains. Markers such as IL-6, hs-CRP, and gait speed are highly relevant to frailty metabolomics, and this consensus framework can guide biomarker qualification [126]. China’s national standard GB/T 44827-2024, which is identical to ISO 23118:2021, has specified pre-examination procedures for metabolomics [127]. Based on these advances, we recommend that international academic organizations such as IAGG or ESPEN lead a multidisciplinary effort to develop the following: first, standardized pre-analytical and data normalization protocols; second, reference ranges for key candidate markers stratified by age, sex, and ethnicity; third, minimum reporting requirements following the STROBE-metabolomics extension; and fourth, a phased regulatory roadmap. Following the ICFSR clinical practice guidelines [128], large-scale longitudinal studies and biomarker-guided intervention trials are needed to move the field towards clinically actionable knowledge.

### 4.3. TMAO in Frailty: Mechanisms and Clinical Translation

Beyond probiotics, prebiotics, and fecal microbiota transplantation, the gut microbiota-derived metabolite trimethylamine-N-oxide (TMAO) has recently been identified as a key mechanistic link between dietary patterns, microbial metabolism, and frailty. This metabolite is generated by gut microbes from dietary quaternary amines such as choline and carnitine [129], with high-protein or high-fat diets being a major source [130]. Mechanistically, TMAO may promote frailty through chronic low-grade inflammation, oxidative stress, endothelial dysfunction, and metabolic dysregulation [131]. Specifically, TMAO exacerbates oxidative stress through mitochondrial dysfunction and reduced antioxidant defenses, triggers endothelial dysfunction and thereby promotes atherosclerosis [132], and activates proinflammatory cascades including the NLRP3 inflammasome [129]. These pathological processes largely overlap with the core pathophysiological features of frailty, suggesting that TMAO may accelerate the onset and progression of frailty through the multisystem involvement of the aforementioned pathways.

Epidemiological evidence further supports a significant association between TMAO and frailty as well as adverse outcomes. A cross-sectional study of 451 older adults with cardiovascular disease showed that each 2 μmol/L increase in plasma TMAO was associated with a 21% higher risk of frailty [133]. More convincingly, data from the community-based Cardiovascular Health Study including 5333 older adults showed that elevated TMAO concentrations independently predicted all-cause mortality [134]. Furthermore, an umbrella review covering 27 systematic reviews and meta-analyses confirmed a positive association between TMAO levels and all-cause mortality, cardiovascular mortality, and stroke risk, although the overall evidence quality was moderate, indicating that more high-quality studies using causal inference methods are needed for validation [135]. However, direct interventions targeting TMAO, including dietary modification, probiotics, and inhibitors of TMAO-producing enzymes, remain largely at the preclinical stage, and their clinical translation has yet to be achieved.

From a clinical translation perspective, microbiota-directed interventions such as probiotics, synbiotics, and fecal microbiota transplantation have shown mechanistic promise in the field of frailty, but their clinical translation remains in an early stage of transitioning from exploratory evidence to standardized protocols. A meta-analysis of 29 randomized controlled trials with 1633 participants confirmed that probiotics and synbiotics can significantly modulate gut microbiota composition, reduce TNF-α levels, and increase short-chain fatty acid production in older adults, thereby providing important evidence for microbiota-based interventions to improve frailty-related outcomes [136]. However, due to substantial heterogeneity in bacterial strains, doses, and intervention durations, further integration of existing evidence is needed to establish standardized protocols. Meanwhile, the number of randomized controlled trials with frailty phenotypes as direct outcome measures is gradually increasing. The RtM trial, registration number NCT07454616, is evaluating the effect of synbiotics combined with exercise on muscle function and the gut microbiome in prefrail adults. The MICROBIOTA trial, registration number NCT07437352, observed the microbiota-modulating effects of probiotics in frail elderly patients receiving home enteral nutrition, providing a basis for subsequent large-scale studies. In the context of fecal microbiota transplantation, the ARMOR trial, registration number NCT06649981, is investigating the effect of oral capsules from young donors on the improvement of functional autonomy in community-dwelling older adults. Preliminary safety is acceptable, but long-term efficacy and regulatory pathways still require further validation. A preliminary randomized controlled trial on butyrate demonstrated that butyrate can repair the intestinal barrier and improve handgrip strength and physical function, offering strong support for short-chain fatty acid-targeted intervention strategies [137]. However, no large-scale trial specifically targeting frail populations has yet been conducted, which provides a clear direction for future research.

In summary, current evidence supports the potential value of TMAO as an intervention target for frailty, but its clinical translation still needs to address issues such as standardization and large-scale validation. Future efforts should prioritize conducting multicenter randomized controlled trials with unified intervention protocols and outcome measures to determine the actual efficacy and target population of TMAO-targeted interventions. Meanwhile, individualized stratification strategies based on TMAO levels can be explored, and multi-omics technologies should be integrated to elucidate its mechanisms of action. Furthermore, microbiota-targeted interventions have made preliminary progress in the clinical translation for frailty. Subsequent efforts should focus on protocol standardization, multicenter randomized controlled trials, and frailty subtype-based precision research to facilitate the translation of evidence into clinical practice.

### 4.4. Limitations

This study has the following four main limitations. First, the data were sourced exclusively from the WoSCC, whose limited coverage may have resulted in the omission of relevant publications. Secondly, inherent methodological differences exist across bibliometric platforms, and their algorithms and text processing capabilities are limited. These platforms typically prioritize predominantly English datasets, which may introduce systematic bias and lead to an underestimation of non-English research contributions and emerging innovations. Moreover, co-citation networks may be influenced by self-citation inflation, where authors disproportionately cite their own work, potentially distorting the representation of influential publications. Thirdly, types of literature including gray literature, such as conference papers and book chapters [138], were excluded, and only peer-reviewed papers and reviews were used at that time. This method may have reduced the scope of the knowledge perspective. Fourth, the keyword standardization process was performed by the authors without independent validation; although a random sample check (10% of the final dataset) showed 100% agreement, subjective decisions in synonym merging and term normalization could introduce bias. Fifth, the study’s time range is from 2006 to 2025; therefore, scholarly output before this period has been excluded, as have the latest publications that have not yet been formally indexed in databases and high-quality recent research with insufficient citation accumulation. The above factors are unable to cover the full range of contextual details, new developments, or other languages. Furthermore, because the WoSCC was queried in early 2026, the 2025 publication data may be incomplete due to indexing delays, and citation counts for recent papers are still accumulating. Sixth, the search strategy focused on “frailty” as the core theme and did not include “sarcopenia” as an independent search term. Although sarcopenia and frailty share considerable pathophysiological overlap, and some of the key sarcopenia-related literature was captured through co-citation analysis, this design may have led to the omission of a very small number of important publications that focus solely on sarcopenia without mentioning frailty. Future dedicated reviews on sarcopenia may adopt a more comprehensive search strategy. Nevertheless, bibliometric analysis remains a valuable tool for systematically comprehending research trends within a field.

## 5. Conclusions

This study provides the first comprehensive analysis of the literature from the past two decades, offering a thorough overview of global research progress and key topics in the field of metabolomics and frailty, and reveals a significant growth in publication output from 2006 to 2025. The United States and China emerged as the primary contributors, with institutions in the United States, such as the University of California System, dominating in terms of both research output and citation impact. Visualization techniques have revealed the extent of interdisciplinary collaborations and identified key journals that have shaped the field. Analysis of research hotspot trends indicates that early studies primarily focused on the associations between macro-level biomarkers and frailty phenotypes, which gradually evolved into the exploration of targeted metabolic pathways and precision-based interventions. The study establishes an evaluation framework for assessing research progress in metabolomics and frailty, and highlights the opportunities for integrating physiology, nutrition, geriatrics, and computational biology.

## Figures and Tables

**Table 1:** Top 10 countries/regions publications.

Rank	Countries /Regions	Np (2006–2025)	Tc (2006–2025)	Average Citation (2006–2025)	H-Index (2006–2025)
1	USA	548	32,752	59.77	89
2	China	304	9375	30.84	39
3	Italy	292	15,340	52.53	61
4	England	177	11,546	65.23	44
5	Japan	150	5980	39.87	37
6	Spain	121	5269	43.55	36
7	Canada	111	7217	65.02	38
8	Australia	94	8183	87.05	34
9	Germany	98	7902	80.63	33
10	Netherlands	78	5699	73.06	32

Note: Np (2006–2025): Total publications in the WoSCC from 2006 to 2025. Tc (2006–2025): The total number of citations of publications from 2006 to 2025 in the WoSCC. Average citation (2006–2025): The average number of citations received per paper from 2006 to 2025. It is a primary indicator for measuring the research quality and impact of different countries or regions, reflecting the average academic influence per paper and facilitating horizontal comparisons across countries or regions with varying publication volumes. H-Index (2006–2025): The H-Index for publications from 2006 to 2025 is an indicator used to measure a scholar’s influence, mainly calculated through the number of articles a researcher has published and the number of citations each article has received, indicating that the scholar has at least h papers that have been cited at least h times.

**Table 2:** Top 10 institutions’ publications.

Rank	Institutions	Country	Np (2006–2025)	Tc (2006–2025)	Average Citation (2006–2025)	H-Index (2006–2025)
1	University of California System	USA	63	5769	91.57	31
2	University of London	UK	60	3348	55.80	24
3	US Department of Veterans Affairs	USA	56	3021	53.95	23
4	Veterans Health Administration (VHA)	USA	53	2827	53.34	22
5	Johns Hopkins University	USA	52	1914	36.81	24
6	National Institutes of Health (NIH)—USA	USA	51	3401	66.69	25
7	Harvard University	USA	45	1559	34.64	19
8	CIBER—Centro de Investigacion Biomedica en Red	Spain	41	2000	48.78	21
9	Catholic University of the Sacred Heart	Italy	40	3135	78.38	26
10	IRCCS Policlinico Gemelli	Italy	39	3014	77.28	26

Note. Np (2006–2025): Total number of articles published from 2006 to 2025. Tc (2006–2025): Total number of citations received from 2006 to 2025. Average Citation (2006–2025): Average citations per article published from 2006 to 2025, calculated as Tc/Np. The H-Index for publications from 2006 to 2025 is an indicator used to measure a scholar’s influence, mainly calculated through the number of articles a researcher has published and the number of citations each article has received, indicating that the scholar has at least h papers that have been cited at least h times.

**Table 3:** Top 10 journals’ publications.

Rank	Journals	Np (2006–2025)	IF (2006–2025)	Partition (2006–2025)	Tc (2006–2025)	H-Index (2006–2025)	Cite Score (2006–2025)	OA (2006–2025)
1	*Clinical Nutrition*	78	7.4	Q1	5684	121	15.5	NO
2	*Nutrients*	73	5.0	Q1	3166	75	9.1	YES
3	*Journals of Gerontology Series a Biological Sciences and Medical Sciences*	68	3.8	Q1	26,387	168	8.9	NO
4	*Geroscience*	62	5.4	Q2	3297	48	8.6	NO
5	*Journal of Cachexia Sarcopenia and Muscle*	36	9.1	Q1	11,230	48	15.6	YES
6	*International Journal of Molecular Sciences*	30	7.3	Q3	444,681	114	9.0	YES
7	*PLoS ONE*	30	2.6	Q3	816,429	268	5.4	YES
8	*Scientific Reports*	28	6.9	Q3	14,436	124	6.7	NO
9	*Aging Cell*	27	7.5	Q3	834,622	149	12.4	YES
10	*BMC Geriatrics*	26	7.9	Q1	19,020	121	6.1	YES

Note: Np (2006–2025) refers to the number of publications a journal has in this specific field. IF (2006–2025) denotes the journal’s impact factor; Partition: JCR quartile classification. TC (2006–2025): Total citations of the journal across all subject areas in the Web of Science Core Collection from 2006 to 2025, derived from the Clarivate Analytics Journal Citation Reports (JCRs) from 2006 to 2025. This value represents the global total citations of the journal and is not limited to the “metabolomics and frailty” topic. H-Index (2006–2025): The cumulative H-Index based on all publications from 2006 to 2025. Cite Score (2006–2025) is the journal citation metric from the database. OA (2006–2025) identifies whether the journal is open access.

**Table 4:** Top 10 authors’ publications.

Rank	Authors	Np (2006–2025)	Tc (2006–2025)	Average Citation (2006–2025)	H-Index (2006–2025)
1	Marzetti, Emanuele	24	1722	71.75	19
2	Calvani, Riccardo	22	1301	59.14	17
3	Walston, Jeremy	22	873	39.68	13
4	Picca, Anna	21	1160	55.24	16
5	Luigi Ferrucci	21	2086	99.33	15
6	Júnior, Hélio Coelho	18	1111	61.72	15
7	Landi, Francesco	18	1877	104.28	17
8	Cesari, Matteo	17	740	43.53	14
9	Rodriguez-Manas, Leocadio	15	1259	83.93	12
10	Sinclair, Alan J.	15	717	47.80	9

Note: Np (2006–2025): Number of publications from 2006 to 2025. Tc (2006–2025): Total citations received from 2006 to 2025. Average Citation (2006–2025): Average citations per publication from 2006 to 2025, calculated as Tc/Np. H-Index (2006–2025): The H-Index for publications from 2006 to 2025, representing the highest number h of publications that have each received at least h citations.

## Data Availability

The original contributions presented in this study are included in the article/Supplementary Materials. Further inquiries can be directed to the corresponding authors.

## Supplementary Materials

The following supporting information can be downloaded at: https://www.mdpi.com/article/10.3390/metabo16060380/s1, Table S1: Search strategy. Our search strategy covered two key concepts: “metabolomics” related terms and “frailty” related terms, combined using the Boolean operator “AND” (see table below). We used this search strategy in the Web of Science Core Collection to identify database specific subject headings. For each search term, we simultaneously searched the abstract, keyword, and title fields, and combined the search with relevant subject headings. The full search strategy was developed with the assistance of a librarian and finalized after peer review. The search platform was the Web of Science Core Collection (WoSCC), and the search date was 6 January 2026; Table S2: The BIBLIO checklist for reporting the bibliometric reviews of the biomedical literature [139,140,141,142,143,144,145,146].
