# Supplementary material for: The Application of Metabolomics in Frailty: Trends, Challenges, and Future Directions
Source: Metabolites. 2026 May 31;16(6):380. doi: 10.3390/metabo16060380 (PMC13302862; doi:10.3390/metabo16060380)
Supplement: Supplementary file 1 [file metabolites-16-00380-s001.zip › Supplementary Materials-Table S1.pdf]

Supplementary Materials: Table S1: Search strategy.

Our search strategy covered two key concepts: “metabolomics” related terms and “frailty” related terms, combined using the Boolean operator “AND” (see table below). We used this search strategy in the Web of Science Core Collection to identify database specific subject headings. For each search term, we simultaneously searched the abstract, keyword, and title fields, and combined the search with relevant subject headings. The full search strategy was developed with the assistance of a librarian and finalized after peer review. The search platform was the Web of Science Core Collection (WoSCC), and the search date was 6 January 2026. All search terms, Boolean operators, and field codes (TS = topic search) are listed below:

(TS=("Metabolomics" OR "Metabolomic"OR "Metabonomics"OR "Metabonomic"OR"metabolize"OR"metabolome"OR"metabolic"OR"metabolsim" OR "metabolon"OR "metabolizing" OR"metabolization"OR "metabolites"OR "metabolite" OR "metabolism" OR "metabolin" OR "metabolically" OR "metabol")) AND (TS=("Frailty" OR "Frailties"OR "Frailness"OR"Frailty Syndrome"OR "Debility"OR"Debilities" OR "Sarcopenias"))

Search term	Keywords
Metabolomics terms	Metabolomics OR Metabolomic OR Metabonomics OR Metabonomic OR metabolize OR metabolome OR metabolic OR metabolsim OR metabolon OR metabolizing OR metabolization OR metabolites OR metabolite OR metabolism OR metabolin OR metabolically OR metabol
Frailty terms	Frailty OR Frailties OR Frailness OR Frailty Syndrome OR Debility OR Debilities OR Sarcopenias
